# Diagnostic Ultrasound Induced Inertial Cavitation to Non-Invasively Restore Coronary and Microvascular Flow in Acute Myocardial Infarction

**DOI:** 10.1371/journal.pone.0069780

**Published:** 2013-07-29

**Authors:** Feng Xie, Shunji Gao, Juefei Wu, John Lof, Stanley Radio, Francois Vignon, William Shi, Jeffry Powers, Evan Unger, E. Carr Everbach, Jinjin Liu, Thomas R. Porter

**Affiliations:** 1 University of Nebraska Medical Center, Omaha, Nebraska, United States of America; 2 Philips Research North America, Briarcliff, New York, United States of America; 3 Philips Medical Systems Inc, Bothell, Washington, United States of America; 4 NuvOx Pharma, Inc, Tuscan, Arizona, United States of America; 5 Swarthmore College, Swarthmore, Pennsylvania, United States of America; S.G.Battista Hospital, Italy

## Abstract

Ultrasound induced cavitation has been explored as a method of dissolving intravascular and microvascular thrombi in acute myocardial infarction. The purpose of this study was to determine the type of cavitation required for success, and whether longer pulse duration therapeutic impulses (sustaining the duration of cavitation) could restore both microvascular and epicardial flow with this technique. Accordingly, in 36 hyperlipidemic atherosclerotic pigs, thrombotic occlusions were induced in the mid-left anterior descending artery. Pigs were then randomized to either a) ½ dose tissue plasminogen activator (0.5 mg/kg) alone; or same dose plasminogen activator and an intravenous microbubble infusion with either b) guided high mechanical index short pulse (2.0 MI; 5 usec) therapeutic ultrasound impulses; or c) guided 1.0 mechanical index long pulse (20 usec) impulses. Passive cavitation detectors indicated the high mechanical index impulses (both long and short pulse duration) induced inertial cavitation within the microvasculature. Epicardial recanalization rates following randomized treatments were highest in pigs treated with the long pulse duration therapeutic impulses (83% versus 59% for short pulse, and 49% for tissue plasminogen activator alone; p<0.05). Even without epicardial recanalization, however, early microvascular recovery occurred with both short and long pulse therapeutic impulses (p<0.005 compared to tissue plasminogen activator alone), and wall thickening improved within the risk area only in pigs treated with ultrasound and microbubbles. We conclude that although short pulse duration guided therapeutic impulses from a diagnostic transducer transiently improve microvascular flow, long pulse duration therapeutic impulses produce sustained epicardial and microvascular re-flow in acute myocardial infarction.

## Introduction

Over one million patients suffer from acute coronary syndromes each year in the United States alone, and over 200,000 of these are ST segment elevation myocardial infarction (STEMI) [1]. Both short and long term prognosis in these patients are improved when there is rapid restoration of flow in the obstructed infarct artery, if it is also associated with restoration of microvascular flow [2]–[5]. Although primary coronary intervention (PCI) of the infarct related artery is the preferred therapeutic option when it can be performed expeditiously, the rapid restoration of epicardial flow is still frequently accompanied by microvascular obstruction [2]. Fibrinolytic therapy has been shown to improve outcome in STEMI, but also is frequently accompanied by microvascular obstruction. Furthermore, the efficacy of full dose fibrinolytic therapy in restoring epicardial flow is only slightly better than 50% [6], [7], and the dose required to achieve this is associated with a higher risk for both intracranial bleeding and major non-cerebral bleeding [8], [9]. Thus, any approach that could reduce fibrinolytic dose yet rapidly restore coronary and microvascular flow would significantly impact short and long term outcome in acute STEMI.

In vitro [10], [11] and in vivo [12]–[15] studies have demonstrated that ultrasound and microbubbles can dissolve intravascular thrombi. When used as an adjunct to thrombolytic agents, ultrasound and microbubbles have resulted in a significant augmentation of the effectiveness of thrombolytics [13]. In previous studies, we have shown that the application of high mechanical index (MI) impulses from diagnostic two-dimensional or three-dimensional ultrasound systems can dissolve thrombi with microbubbles, even with transthoracic attenuation of the ultrasound impulses [16], [17]. Microbubble cavitation has been considered as the presumptive mechanism for enhanced thrombus dissolution. The rapid growth and collapse of microbubbles during inertial cavitation results in increased shear stress along the thrombus, and more fluid jets that penetrate the thrombus [18], [19]. High speed optical observations have demonstrated that these phenomena occur in microseconds, following which the gas nuclei dissolve and the cavitation ceases [19]. Recent in vitro studies have indicated that the inertial cavitation induced by a therapeutic impulse can be sustained with a longer pulse duration. Although most diagnostic ultrasound impulses are shorter in duration (5 usec) to preserve spatial resolution, we hypothesized that therapeutic impulses which sustain an inertial cavitation response (i.e. longer pulse duration) would more likely produce greater thrombus dissolution in a clinically relevant in vivo setting. To test this hypothesis, we modified a conventional diagnostic imaging system so that it produced a longer pulse duration therapeutic ultrasound (TUS) impulse. This was then tested during an intravenous microbubble infusion to determine a) whether it was capable of inducing inertial cavitation within the myocardium and b) whether it could restore epicardial and microvascular flow in an established animal model of acute myocardial infarction.

## Methods

### Ethics Statement

All animal studies were compliant with the standards in the National Research Council’s Guide for the Care and Use of Laboratory Animals and approved by the University of Nebraska Medical Center (UNMC) Institutional Animal Care and Use Committee (permit number: 08-069). UNMC has been accredited by the Association for Assessment and Accreditation of Laboratory Animal Care (AAALAC) continuously since 1966. All procedures were performed under isoflurane anesthesia and all efforts were made to minimize suffering by the experimental animals.

### Microbubbles

The MRX-801 microbubbles (NuvOx Pharma, LLC, Tucson, Arizona) used for this study are lipid encapsulated microbubbles of a perfluoropropane gas, and have a diameter of 1.0±0.1 µm, with a concentration of 1.5 to 3.0×10^10^/ml. The microbubble infusions for the study were prepared by diluting 2 ml of the MRX-801 in 100 ml of 0.9% saline and infusing at a rate of 2.5–3.0 ml/min.

### Diagnostic Ultrasound System Modification

**Figure 1:**
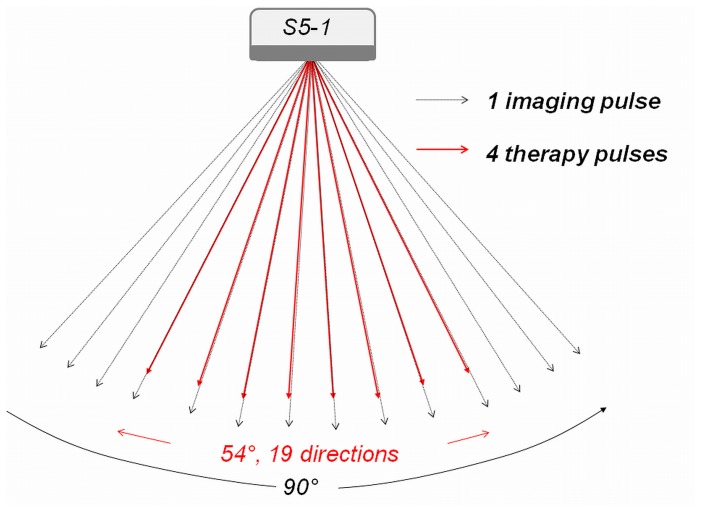
Depiction of the Modified Transducer for Thrombolysis. Schematic depiction of the modified diagnostic ultrasound transducer on the Philips S5-1 scanner. The therapeutic impulses were sent as ensembles of four pulses of the same length, while shorter B-mode pulses of lower amplitude were interleaved for background images of microbubble replenishment within the risk area.

An ultrasound system (iE33; Philips Healthcare, Andover, MA) was modified for image-guided sonothrombolysis capability (Figure 1). The specific modifications included: (a) a therapeutic ultrasound (TUS) mode with a pre-defined treatment area (similar to the box in color Doppler imaging) superimposed on the anatomic imaging mode (B-mode); and (b) a low MI contrast-only imaging mode for monitoring the presence (replenishment) of microbubbles. For each TUS frame, 19 equally spaced beams (each with an ensemble of four 1.6 MHz long pulses) were scanned into a 54 degree angular sector area [20]. The beams were focused at the center of the risk area. The frame rate for the TUS mode was set to be around 50 Hz. The acoustic energy for the imaging pulses in the background B-mode was much less that for the two TUS settings, so the therapeutic contribution from the B-mode was negligible. Once echo signals on the low MI contrast mode (0.1 MI) reached a plateau intensity in the risk area, the TUS modes with predetermined MI (2.0 and 1.0) and pulse length (5 and 20 µs respectively) were activated and TUS scanning of the risk area proceeded from apex to base (approximately four seconds).

### Analysis of Induced Cavitation States

In order to determine whether intra-myocardial inertial cavitation was occurring during the two different TUS impulses used in this study, an open chest preparation was created in two pigs via a lateral thoracotomy. The heart was exposed and placed in a pericardial cradle. A three centimeter thick tissue mimicking phantom was placed over the anterior epicardium to simulate transthoracic attenuation. A 20 MHz transducer (used as a passive cavitation detector) was placed underneath the tissue mimicking phantom confocal to the imaging and therapeutics transducer to record if inertial cavitation (defined as high amplitude spikes from the 20 MHz passive transducer) was occurring with either of the TUS impulses. All measurements were analyzed during the same continuous infusion of 3% MRX-801 microbubbles used for subsequent randomized treatments. In order to confirm that the cavitation signals were coming from the anterior myocardium (and not the adjacent LV cavity), the oscilloscopic tracings of scattered signals from the anterior myocardium were measured before, during and after LAD balloon occlusion.

### Animal Preparation For Closed Chest Studies

The protocol involved 36 atherosclerotic pigs who developed coronary atherosclerotic lesions using a previously described protocol [17]. On day one, domestic swine were anesthetized with isoflurane anesthesia. An 8F guide catheter was introduced through a femoral arterial line into the left main ostium. Following a 100 microgram injection of intracoronary nitroglycerin, baseline angiograms were obtained and the mid left anterior descending (LAD) sized with a digital caliper. A balloon catheter which measured 10% larger than the vessel size was then inflated in the LAD to create endothelial injury. Following this, the pigs were recovered and placed on a high saturated fat-cholesterol diet for 50 days (15% Lard, 2% Cholesterol; Harlan Laboratories, Madison, WI).

### Experimental Study Protocol

At approximately 50 days following balloon injury, the pigs were re-anesthetized. Two femoral artery and venous catheters were placed for hemodynamic monitoring and infusions of microbubbles. A 6F pigtail catheter was inserted into the left atrium via one arterial line to monitor left atrial pressures. Coronary artery thrombotic occlusions were created at the same LAD site by re-injuring the original site of balloon inflation with an over-sized balloon. Specific angiographic markers were used (e.g. location in distance from a diagonal branch) to ensure that injury was at the same location as day one. After re-injury, the balloon was withdrawn proximal to the injury site and partially inflated to create vessel stasis. Following this, small 0.1–0.2 milliliter aliquots of thrombosed venous blood (obtained from the same animal) were introduced through the partially inflated balloon catheter to create a hypercoagulable state. Once angiographic LAD occlusion was documented, it had to persist for at least 20 minutes before randomization. If spontaneous recanalization occurred, additional 0.1–0.2 milliliter aliquots of thrombus were again administered through the balloon catheter until persistent occlusion was observed.

The percent oxygen mixture was kept at 24% during treatment periods in all pigs. Low-dose intravenous dobutamine (1 to 3 ug/kg per minute) was used, if necessary, to maintain systolic arterial pressure >90 mm Hg during the study protocol. Lidocaine boluses (40 mg IV followed by 20 mg IV boluses ×3) and a continuous lidocaine infusion (2 to 4 mg/min IV) were used in all animals to control arrhythmias. Direct current cardioversions (300 Joules) were used, as necessary, to treat sustained ventricular tachycardia or ventricular fibrillation.

**Figure 2:**
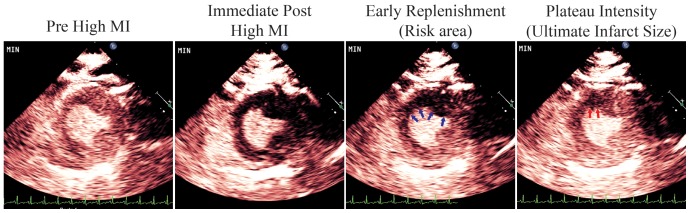
Myocardial Perfusion Images. Myocardial perfusion images from the low MI contrast imaging that were used to guide the application of the therapeutic ultrasound (TUS) impulses. Note that after the TUS impulses, there was no myocardial contrast present in the left circumflex or left anterior descending perfusion beds (Second panel from the left). During low MI triggered imaging, early replenishment delineated the risk area (arrows; third panel from the left). A plateau intensity was reached (right panel), which was when the TUS impulses were re-applied.

Just prior to randomized treatment, pigs had baseline measurements of heart rate, oxygen saturation, arterial blood pressure, and left atrial pressure. Wall thickening and perfusion defect size (see below) were determined during a 3% intravenous microbubble infusion (MRX 801; NuVox Pharma; Tuscon, Arizona) using low MI imaging (0.2 MI; Power Modulation; Philips S5-1). Maximum ST segment elevation was measured with a 12 lead EKG. Subsequently, all pigs received 650 milligrams of aspirin per a nasogastric tube, followed by an intravenous heparin bolus (80 mg/kg), and a low dose of fibrinolytic agent (0.5 mg/kg tissue plasminogen activator administered over 30 minutes). The pigs were randomized into three groups: no additional treatment (n = 12; referred to as Group I); a continuous intravenous infusion of MRX-801 with intermittent 2.0 MI short duration TUS impulses (1.6 MHz; 5 usec pulse duration, subsequently referred to as Group II, N = 12) and intermittent 1.0 MI longer duration TUS impulses (1.6 MHz; 20 usec pulse duration; Group III, N = 12). The lower MI was used for the longer TUS pulse duration to match the energy outputs in Groups II and III. TUS impulses were administered for five seconds while manually scanning the anterior myocardium from apex to base. In between TUS pulses, low MI imaging (Power Modulation) was used with end-systolic triggering to detect for microbubbles within the risk area, which was defined as the region exhibiting a contrast defect during the replenishment phase of contrast following therapeutic impulses (Figure 2). All treatment durations were 30 minutes. Venous samples for activated clotting times (ACTs) were obtained before initiation of treatment and at approximately 30 minutes into randomized treatments.

### Outcome Measurements

In all pigs, epicardial recanalization was assessed by angiography using left main coronary artery injections of five milliliters of iodinated contrast at 30, 60, and 90 minutes following initiation of treatment protocols. Twelve lead EKG’s were obtained at baseline prior to treatment, and at 30, 60, and 90 minutes into each randomized treatment. Maximal ST segment elevation, and % ST segment resolution from maximum, were compared at each time point. Wall thickening within the central portion of the risk area on a two dimensional short axis view (mid-papillary muscle level) was computed from off line from end-diastolic and end-systolic wall thickness measurements prior to treatment, and at sixty minutes into treatment. Percent wall thickening was defined as the difference in thickness measurements at these two time points divided by end-diastolic wall thickness, x100% [16].

The risk area was approximated by defining the circumferential extent of the perfusion defect during the early phase of contrast replenishment following a high MI impulse during LAD occlusion [21]. To determine ultimate infarct size, planimetered measurements of end-systolic defect size (hypoperfused area) were obtained at plateau intensity at 90 minutes into treatment during low MI imaging and a continuous infusion of microbubbles [21]. All measurements were obtained by an experienced reviewer (JW). Inter -observer correlations for wall thickening and perfusion defect size at plateau intensity were also computed by comparing the correlation of 126 wall thickness and 48 perfusion defect size measurements obtained by second reviewer (JW).

### Post Mortem Measurements

After the 90 minute angiograms, the pigs were sacrificed. The LAD coronary artery was dissected and analyzed with Hematoxylin/Eosin staining for presence of intra-coronary thrombus, focal dissection, and evidence of underlying atherosclerosis, as determined by the presence of fatty streaks and intimal hyperplasia. Percent vascular obstruction by the underlying plaque was visually graded as 0–25%, 25–50%, and 50–75% using established criteria [22]. The LAD proximal to the balloon injury site was examined for evidence of endothelial damage or hemorrhage that may have been as a result of the high MI impulses. All analyses here were done by an experienced cytopathologist (SJR) blinded to treatment assignment.

Prior to sacrifice in nine pigs, Evan’s Blue was injected into the right and left coronary arteries during the mid left anterior descending balloon occlusion (at the site of the original injury) to correlate risk area, determined by this stain, with myocardial contrast echocardiographic defect size during the early replenishment phase of contrast immediately following high MI impulses.

### Statistical Analysis

Hemodynamic and echocardiographic changes within groups before and after treatment, as well as activated clotting time measurements before and after treatment, were compared using paired t testing. Comparisons of recanalization rates between the three treatment groups were compared using contingency tables. Comparisons of wall thickening changes, ST segment changes, and changes in plateau intensity perfusion defect size between groups were perfomed using analysis of variance. The relationship between perfusion defect size during the early replenishment phase contrast following high MI impulses and actual risk area measured with intracoronary Evan’s Blue injections during LAD occlusion were determined with a Pearson correlation coefficient. In all cases, p values <0.05 were considered significant.

## Results

### Cavitational Analysis of Therapeutic Impulses

**Figure 3:**
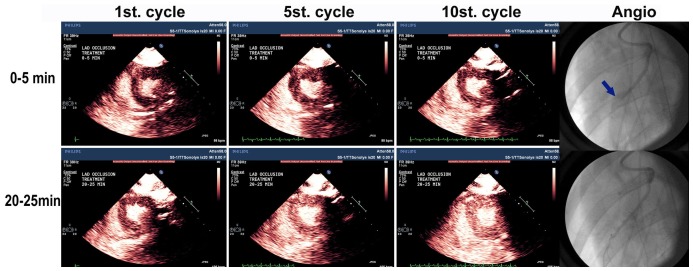
Intra-myocardial Passive Cavitation Detector Recordings During Therapeutic Impulses. Passive cavitation detector recordings from the anterior myocardium during a continuous infusion of microbubbles and application of the long pulse duration 1.0 MI (left panel) and short pulse 2.0 MI (right panel) therapeutic impulses. The black dots above the baseline signal represent inertial cavitational activity, which disappeared when the left anterior descending was occluded (The “TUS On” following balloon inflation), confirming that recorded activity was intra-myocardial. Note that the amplitude of the voltage, and number of recorded events produced by the cavitational spikes were similar for the two different therapeutic impulses. However, since the 20 usec pulse lasts four times longer than the 5 usec pulse, the aggregate cavitational activity for the 20 usec pulse is nearly four times longer (0.73 volt-seconds versus 0.22 volt-seconds).

**Figure 4:**
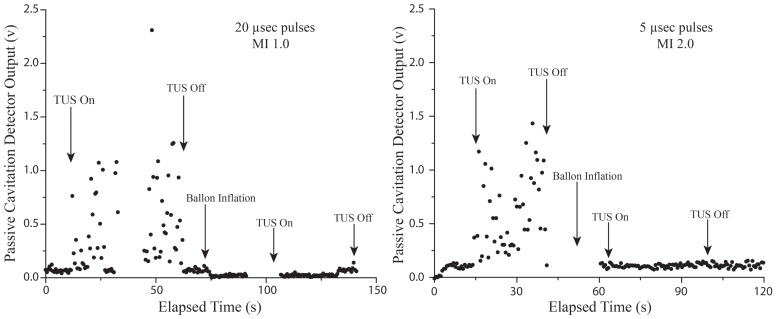
Microvascular and Angiographic Recanalization with Echo Guided Long Pulse Duration Therapeutic Impulses. The angiographic results in one of Group III pigs treated with guided long pulse duration TUS impulses. The short axis echocardiographic images delineate myocardial contrast enhancement at incremental cardiac cycles following the therapeutic impulse. The cycle number represents the number of cardiac cycles that have occurred following cessation of the TUS impulses. Although there was minimal capillary replenishment noted at 0–5 minutes into therapy, at 20–25 minutes there was more rapid restoration of contrast enhancement within the LAD risk area. Note angiographic recanalization was observed, (right lower panel), as well as improved microvascular perfusion (as indicated by the rapid replenishment of microbubbles into the risk area) at 20–25 minutes into therapy.

Cavitation signals received by the passive cavitation detector in the open chest pigs during application of the two different applied TUS impulses are shown in Figure 3. Note that inertial cavitation (the signal spikes measured on the oscilloscope) was present during the application of both the short and long TUS impulses used in the study. There were no differences in the amplitude of cavitational activity (which indicates the magnitude of growth and collapse of the microbubbles) between the 2.0 MI short pulse duration impulses and the 1.0 MI longer pulse duration impulses. However, since the 20 usec pulse lasts four times longer than the 5 usec pulse, the aggregate cavitational activity for the 20 usec pulse was nearly four times longer (0.73 volt-seconds versus 0.22 volt-seconds). These inertial cavitation signals were confirmed to be coming from the anterior myocardium, as they almost completely disappeared during LAD occlusion, and reappeared when applied following reperfusion of the LAD (Figure 4).

### Hemodynamic Comparisons

Table 1 summarizes the hemodynamic, heart rate, and oxygen saturation measurements following coronary occlusion just prior to treatment, and at sixty minutes following initiation of treatment. There were no differences between groups in any of these parameters before or after randomized treatments, except activated clotting times due to heparin. The amount of thrombus required to occlude the LAD was 0.5±0.1 ml in Group I, 0.5±0.2 ml in Group II, and 0.5±0.2 ml in Group III. No ventricular arrhythmias were noted during application of either of the TUS impulses at any stage of the 30 minute treatment period.

**Table 1:** Hemodynamic Characteristics.

	Group I		Group II		Group III	
	Before	After	Before	After	Before	After
Heart rate (beat/minute)	96±14	100±11	95±13	101±11	93±15	105±16
Systolic arterial pressure(mmHg)	107±12	105±9	105±9	99±11	105±7	99±11
iastolic arterial pressure(mmHg)	73±10	69±15	76±12	67±12	72±11	65±14
SpO^2^ (%)	100±0	100±1	100±0	100±0	100±0	100±0
Left atrial pressures(mmHg)	7±4	6±3	8±1	7±4	8±4	8±3
ACT (s)	114±14	187±19*	112±12	181±25*	115±13	187±20*

* <0.001 compared to before treatment.

### Angiographic, Electrocardiographic, and Wall Thickening Measurements

The pigs in group III had an 83% angiographic recanalization rate at 30, 60 and 90 minutes (compared to 42%, 42% and 45% for Group I pigs or TPA alone, respectively; p<0.05). Recanalization rates for group II (short pulse TUS impulses) were 58% at these same time periods. Figure 3 is an example of a coronary angiogram of a recanalized left anterior descending artery after 30 minutes of treatment with the long duration 1.0 MI TUS impulse, as well as the improvement in myocardial perfusion within the risk area after treatment. In both Group II and III pigs, there was more rapid replenishment of microbubbles within the risk area following high MI impulses, as treatment time progressed (Figure 3).

Both short (Group II) and long (Group III) pulse TUS treated pigs exhibited improved microvascular flow by EKG. ST segment change improved 60% at 30 minutes into treatment in group III, compared to 48% in group II and 10% in group I (P<0.05 for both Group III and Group II *vs*. Group I). ST segment resolution >50% at 60 minutes into treatment occurred in 10 of 12 pigs (83%) in Group III, eight of 12 pigs (67%) in group II, and four of 12 pigs (33%) in Group I (P = 0.006). Wall thickening within the risk area at 60 minutes was significantly improved in Group III and Group II pigs compared to Group I.

Plateau myocardial contrast defect size improved only in Groups II and III pigs (Table 2). Contrast defect size improved even in the pigs that did not exhibit epicardial recanalization (p = 0.003), but not in the pigs treated with TPA alone.

**Table 2:** Wall thickening and perfusion defect size (at plateau intensity) before and following treatment.

	Group I	Group II	Group III
Baseline WT	8±5%	9±7%	11±8%
60 minutes WT	11±9%	25±8%	19±12%
WT % Improvement	3±5%	15±12%*	8±10%*
Baseline PD Size	1.74±0.69 cm^2^	1.67±0.59 cm^2^	1.71±0.65 cm^2^
60 minutes PD Size	1.48±0.35 cm^2^	1.19±0.62 cm^2^ *	0.84±0.51 cm^2^ *

* p<0.05 compared to Group I. Group I = 1/2 dose TPA alone; Group II = 1/2 dose TPA with guided short pulse duration TUS impulses; Group III = 1/2 dose TPA with guided long pulse duration TUS impulses.WT =  wall thickening; PD = perfusion defect.

Interobserver correlation in measurements of wall thickening and perfusion defect size were r = 0.96 (n = 126 comparisons) and r = 0.91 (n = 48 comparisons), respectively. In the nine separate pigs used to validate risk area measurements, there was a close correlation between risk area calculated by contrast perfusion defect size during early replenishment following high MI impulses measured with MCE in the initial phases of treatment, and that seen with Evans Blue staining during LAD occlusion (r = 0.82, p<0.005).

### Post Mortem Coronary Artery Measurements

Post mortem pathologic analysis of the LAD was not possible in two pigs. Evidence of early atherosclerotic lesions within the balloon injured portion of the LAD was evident in 31 of the remaining 34 pigs (91%). In 25 of these pigs (74%), the obstruction created by plaque was <25%, while in six it was either 25–50% (n = 3) or 50–75% (n = 3) in severity. In three pigs, there was no evidence of plaque formation at the site of injury. There was evidence of focal intimal dissection (as a result of the balloon injury) in 19 pigs (56%). No differences were observed between treatment groups in the presence or absence of any of these parameters.

## Discussion

This prospective randomized pre-clinical study has demonstrated that a modified diagnostic ultrasound system can be utilized with intravenous microbubbles and a low dose of TPA to rapidly restore epicardial and microvascular flow in acute STEMI. The TUS impulses that improved both epicardial and microvascular flow were of longer duration than those used for diagnostic imaging, but still at an MI that would still be considered within Food and Drug Administration limits. We also documented that this longer pulse duration TUS impulse induced inertial cavitation within the microvasculature, and, unlike TUS impulses of shorter duration, was able to recanalize the epicardial vessels as well as the microvasculature. The key mechanistic difference between the two TUS impulses tested was the ability of the long pulse duration to sustain the cavitation effect for longer microsecond intervals.

Thrombotic coronary occlusions produce downstream microthrombi, which are believed to play a significant role in the no-reflow phenomena [23]. This microvascular obstruction prevents restoration of function within the risk area, and can persist even after epicardial recanalization. Previous studies have demonstrated improved microvascular perfusion to the risk area with short pulse transthoracic ultrasound-induced microbubble cavitation, even without early epicardial recanalization [16], [17]. One mechanism for this microvascular improvement, in the absence restored anterograde blood flow, appears to be ultrasound induced endothelial nitric oxide release, resulting in vasodilation and improved flow from collateral vessels [24], [25]. In our study, the TUS impulses did improve microvascular function acutely even without epicardial recanalization, as evidenced by ST segment resolution, reductions in perfusion defect size, and early improvements in wall thickening within the risk area. These findings are consistent with studies examining improvements of tissue perfusion in the presence of persistent upstream occlusion. However, in the absence of epicardial recanalization, these improvements were present primarily when the ultrasound was applied, and reversed when ultrasound was withdrawn. Therefore, it would appear critical that any therapeutic technique in this setting should also restore anterograde epicardial blood flow. In this context, the guided long pulse TUS impulses would have the advantage over current therapies, in that they can both maintain microvascular function and rapidly restore epicardial flow.

The in vivo inertial cavitation measurements demonstrated that longer pulse duration therapeutic impulses at a lower MI may have beneficial mechanistic effects over high MI shorter duration impulses, because they prolong the duration of induced cavitation. This may have several clinical advantages as well. Since the inertial cavitation with the longer pulse was achieved with a lower MI, it may be less likely to cause any unwanted bioeffects, such as arrhythmias or myocyte damage [26]. Secondly, the sustained cavitation induced by the longer pulse duration could potentially result in more exposure of plasmin binding sites for the co-administered low dose plasminogen activator [27]. Thirdly, sustaining inertial cavitation with the longer pulse duration would result in more fluid jets from cavitating microbubbles, and improve the likelihood of more rapidly mechanically dissolving the thrombus in both the epicardial vessel and the microvasculature.

The augmentation of thrombolysis with ultrasound and microbubbles observed in this study is similar to what has been observed in animal models of acute thrombo-embolic stroke, where transtemporal ultrasound and microbubbles alone have reduced stroke size similar to what full dose TPA (0.9 mg/kg) achieved [28]. In these animal studies, a longer 1 Megahertz pulse (20% duty cycle) at a lower mechanical index was utilized to cavitate microbubbles. By reducing or even eliminating the need for fibrinolytic therapy, ultrasound and microbubbles could expand the number of patients in each of these clinical settings who are eligible for therapy, especially those who are at increased risk for bleeding (e.g. elderly patients or patients with prior stroke). In the setting of acute stroke in animal models, the utilization of ultrasound and microbubbles alone has reduced infarct size, and been shown to reduce the frequency of intracranial hemorrhage when compared to fibrinolytic therapy [29].

### Limitations of the Study

Although the majority of pigs in this study developed some degree of early atherosclerotic plaque within the balloon injured area, the severity of underlying plaque was less than 50%. It is unclear how effective the long pulse duration TUS impulses would be when more advanced forms of atherosclerosis and calcification are present. Furthermore, the thrombotic occlusion was fresh and less than two hours old in the model tested, and thus it is unknown what results would be expected if the time interval between occlusion and treatment was longer than this.

We only tested one longer pulse duration TUS impulse, in order to stay close to Food and Drug Administration limits. It is possible that even longer pulse durations than those used in this study may be more effective for thrombolysis. However, longer pulse durations may increase the risk for heating in low flow areas, which may be counterproductive in this setting [30], [31]. This may require alterations in transducer design, such as capacitive micromachined ultrasound transducers, which do not have as much self-heating problems as piezoelectric transducers [32].

Finally, we did not test what effect stable cavitation alone may have in this setting, since inertial cavitation was induced by both of the TUS impulses. Although stable cavitation is effective in improving the effectiveness of fibrinolytic therapy in vitro, in vivo studies have demonstrated that the inertial cavitation threshold must be reached before effective intravascular thrombolysis occurs [33].

### Clinical Implications

The model chosen for this study emulates the setting in which most myocardial infarctions occur clinically. Usually thrombotic occlusions occur from ruptured plaques that were previously non-obstructive [34], [35], resulting in coronary occlusions that are composed of predominately thrombus. Underlying plaque was present in the majority of pigs in this study, but was <25% diameter in over 70% of pigs. In this setting, the 83% epicardial recanalization rate achieved with the long pulse duration TUS impulses is comparable to that seen with interventional percutaneous therapies. Moreover, since echocardiography is more portable and readily available to the patient than interventional laboratories, this therapy could reach a higher number of patients more rapidly, resulting in greater degrees of myocardial salvage in acute STEMI.

The long pulse duration TUS impulses used in this study were administered from a modified commercially available diagnostic transducer, and the intravenous microbubbles were very similar to the commercially available microbubble Definity (Lantheus Medical). The TUS impulses also did not induce arrhythmias when they were applied, indicating the TUS impulses were not causing unwanted bioeffects within the coronary artery. Furthermore, the TUS impulses, even if they did not restore epicardial flow, did have a temporary effect of improving microvascular flow, and thus may be able to salvage myocardium until epicardial flow is restored by interventional techniques. Therefore, this approach may be effective, with or without low dose TPA, in a clinical setting where emergent percutaneous therapy is not feasible, or where there may be increased bleeding risk associated with higher doses of TPA. With guided long pulse duration TUS impulses, one could non-invasively achieve epicardial recanalization rates that approach that seen with interventional therapies, and, more importantly, overcome the problem of persistent microvascular obstruction that complicates current state of the art therapies.
